# Serum Can Overcome Contact Inhibition in Confluent Human Pulmonary Artery Smooth Muscle Cells

**DOI:** 10.1371/journal.pone.0071490

**Published:** 2013-08-06

**Authors:** Victor Solodushko, Heba A. Khader, Brian W. Fouty

**Affiliations:** 1 Center for Lung Biology, University of South Alabama School of Medicine, Mobile, Alabama, United States of America; 2 Department of Pharmacology, University of South Alabama School of Medicine, Mobile, Alabama, United States of America; 3 Department of Medicine/Division of Pulmonary and Critical Care Medicine, University of South Alabama School of Medicine, Mobile, Alabama, United States of America; University of Sassari, Italy

## Abstract

Pulmonary artery endothelial cells (PAEC) in an intact vessel are continually exposed to serum, but unless injured, do not proliferate, constrained by confluence. In contrast, pulmonary artery smooth muscle cells (PASMC) attain, and maintain, confluence in the presence of minimal serum, protected from serum’s stimulatory effects except when the endothelial barrier becomes more permeable. We hypothesized therefore, that confluent PASMC may be less constrained by contact inhibition in the presence of serum than PAEC and tested this idea by exposing confluent non-transformed human PAEC and PASMC to media containing increasing concentrations of fetal bovine serum (FBS) and determining cell growth over 7 days. PAEC that had attained confluence in low serum did not proliferate even when exposed to 5% serum, the highest concentration tested. In contrast, PASMC that attained confluence in low serum did proliferate once serum levels were increased, an effect that was dose dependent. Consistent with this observation, PASMC had more BrdU incorporation and a greater percentage of cells in S phase in 5% compared to 0.2% FBS, whereas no such difference was seen in PAEC. These results suggest that confluent human PAEC are resistant to the stimulatory effects of serum, whereas confluent PASMC can proliferate when serum levels are increased, an effect mediated in part by differences in phosphoinositide 3-kinase activation. This observation may be relevant to understanding the PASMC hyperplasia observed in humans and animals with pulmonary hypertension in which changes in endothelial permeability due to hypoxia or injury expose the underlying smooth muscle to serum.

## Introduction

Contact inhibition, the arrest of growth induced by confluence, is characteristic of most normal cells and cell lines and is important in preventing excessive neoplastic and non-neoplastic proliferation. Multiple pathways important in mediating contact inhibition have been identified in different cell types and under different conditions. [1]–[7] It is not clear whether these different pathways are exclusive to a particular cell type or whether multiple pathways may be active in cells at any time. In addition, it is not clear whether any particular pathway may be more effective than another at inducing contact inhibition in the face of continuous exposure to growth factors. Contact inhibition to prevent non-neoplastic proliferation is perhaps most important in vascular endothelial cells since they are continually exposed to growth factors and serum. In contrast, vascular smooth muscle cells are not directly exposed to serum, shielded by overlying endothelial cells. As a result, vascular smooth muscle cells may have less robust mechanisms for enforcing contact inhibition following exposure to serum than vascular endothelial cells.

Pulmonary artery smooth cell (PASMC) proliferation is an important pathophysiologic event in the development of pulmonary hypertension. While the mechanisms leading to PASMC hyperplasia in pulmonary hypertension are not entirely clear and may vary according to the initiating insult, [8]–[10] exposure of the underlying PASMC to serum due to endothelial injury or increased permeability, may be an important stimulus. If contact inhibition in PASMC is as strictly enforced as it is in pulmonary artery endothelial cells (PAEC), however, exposure of contact-inhibited PASMC to increased serum concentrations and growth factors should not be sufficient to stimulate proliferation.

To test the hypothesis that acute exposure to increased serum levels would stimulate confluent PASMC, but not confluent PAEC, to proliferate, we grew human cells from the pulmonary circulation, allowed them to achieve confluence in low serum, and then exposed them to increasing doses of serum.

## Methods

### Materials

SmBM (Smooth Muscle Cell Basal Medium, CC-3181) with SmGM-2 SingleQuots (CC-4149) and EBM (Endothelium Cell Basal Medium, CC-3121) with EGM-MV SingleQuots (CC-4143) were from Lonza (Walkersville, MD). DMEM, propidium iodide, RNase, and 5′-bromo-2′-deoxyuridine (BrdU), LY294002, Wortmannin from Penicillium funiculosum (W1628), and bovine serum albumin (A7030) were all from Sigma (St. Louis, MO). Trypsin-EDTA and L-glutamine were from Gibco (Grand Island, NY). FBS was from Atlanta Biologicals (Lawrenceville, GA). HyBond-P membrane was from Amersham (Buckinghamshire, England). Ethidium homodimer-1 (L3224) was from Molecular Probes. SuperSignal West Dura (34076) and SuperSignal West Femto (34036) were both from Pierce (Rockford, IL).

### Antibodies Used

Cyclin D1 (DCS-6) was from Santa Cruz Biotechnology (Santa Cruz, CA). p27^Kip1^ (13231A) and Rb (554136) were from PharMingen (San Diego, CA). β-actin peroxidase (A3854) was from Sigma, and BrdU (555627) was from BD Biosciences, San Diego, CA. AKT (9272), phospho-AKT (Ser473) (4058), and phospho-AKT (Thr308) (4056) all were from Cell Signaling. Secondary horseradish peroxidase-conjugated antibodies used were sheep anti-mouse (NA931V) and goat anti-rabbit (RPN4301) from GE Healthcare UK Limited (Little Chalfont Buckinghamshire, UK).

### Cell Culture

Human Pulmonary Artery Endothelial cells (PAEC) and Human Pulmonary Artery Smooth Muscle cells (PASMC) were purchased from Cambrex (Walkersville, MD) and used at passage 4 to 7. Human PASMC were cultured in SmBM (Smooth Muscle Cell Basal Medium, Lonza, CC-3181) supplemented with SmGM-2 SingleQuots (Lonza, CC-4149). Human PAEC were cultured in EBM (Endothelium Cell Basal Medium, Lonza, CC-3121) supplemented with EGM-MV SingleQuots (Lonza, CC-4143). For some experiments Human PASMC and PAEC were cultured with reduced or elevated serum level or in the presence of PI3K inhibitors: LY294002 (15 µM) or Wortmannin (50 nM). Rat PAEC and rat PASMC were isolated and characterized in our cell culture core laboratory. [11] We used these cells in parallel to the human cells. Rat cells were routinely cultured in DMEM/10% FBS and used for the experiments at passage 4 to 7.

### Cell Proliferation

All cells were grown in humidified incubators at 37°C in 5% CO_2_ and then exposed to experimental conditions. Cells were harvested by 0.05% trypsin/0.53 mM EDTA digestion and counted with Coulter Z1 (Coulter Electronics). Counts were made in triplicate.

### Western Blotting

Cells were harvested and lysed in RIPA buffer (150 mM NaCl, 10 mM Tris, pH 7.2, 0.1% SDS, 1.0% Triton X-100, 1% deoxycholate, 5 mmol/l EDTA, 1 mmol/l phenylmethylsulfonyl fluoride, 10 mmol/l benzamidine, 10 µg/ml leupeptin, 10 µg/ml aprotinin), incubated on ice for 10 min, and centrifuged at 13,000 *g* to clear the lysates. Protein content from total cell lysates was determined by Bradford assay. Proteins were resolved in SDS-PAGE and transferred to PVDF membranes. Membranes were incubated in blocking solution and probed with primary antibodies. Positive antibody reactions were visualized using peroxidase-conjugated secondary antibodies and a SuperSignal chemiluminescence detection system (Pierce) according to the manufacturer’s instructions.

### Cell Cycle Analysis

Cells were digested with trypsin-EDTA from culture plates, and the trypsin was inactivated by addition of 10% FBS, washed with PBS by low centrifugation, and incubated in Krishan’s solution [50 µg/ml propidium iodide (PI), 0.1% sodium citrate, 20 µg/ml RNase A, 0.3% Ipegal overnight at 4°C.] Cells were analyzed directly by fluorescence-activated cell sorting using FACScan in the University of South Alabama Flow Cytometry Core.

### BrdU Incorporation

For BrdU labeling, cells were pulse treated for 10 min with 10 mmol/l BrdU, washed three times with PBS, and harvested as described above. Cells in 200 µl PBS were slowly added to 5 ml of 70% ethanol (–20°C) while maintaining a vortex. After 30 min of incubation (fixation), cells were collected by centrifugation (500 *g*, 10 min, 10°C). One ml of 2N HCl/Triton X-100 was slowly added to the cells. After a 30-min incubation at room temperature (to produce single-stranded DNA), cells were collected and re-suspended in 1 ml of 0.1M Na_2_B_4_O_7_×10H_2_O, pH 8.5, to neutralize acid and were collected again. Cells were incubated with an anti-BrdU antibody for direct immunofluorescence staining. The samples were analyzed by FACScan.

### Adenoviral Infection

Human PASMCs and PAECs were infected for 2 hours at a multiple of infectivity of 200 with a replication-deficient adenovirus serotype 5 containing a human p27^KIP1^ cDNA driven by a CMV promoter [12], [13]. An adenovirus encoding the human placental alkaline phosphatase cDNA was used as a control.

### Cytotoxicity Assay

Cell monolayers were pretreated for 20 minutes with 4 µM Ethidium homodimer-1 (2 mM stock in DMSO), washed in PBS and analyzed on a fluorescence plate reader (Fluoroscan Ascent; Labsystems, Finland) using a 530 nm excitation and 620 nm emission filter pair.

### Statistical Analysis

Data are expressed as means ± SE. Cell growth and changes in cell cycle profile were compared using ANOVA combined with Fisher post hoc analysis, with a *P* value <0.05 considered significant. BrdU incorporation, cytotoxicity data, and protein expression/phosphorylation levels, were compared using a two-tailed unpaired *t*-test with a *P* value <0.05 considered significant.

## Results

### Confluent PASMC, but not PAEC, Proliferate in Response to Increased Serum

**Figure 1:**
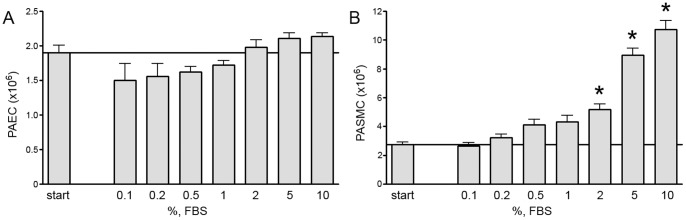
Exposure to increased serum stimulates growth in confluent PASMC, but not in confluent PAEC. Human PAEC (A) and PASMC (B) attained confluence in low (0.2%) serum as determined by light microscopy and the absence of proliferation. Cells were then exposed to increasing concentrations of serum and cell number determined in triplicate seven days later. (n = 3 experiments; * indicates p<.05 compared with starting cell number).

**Figure 2:**
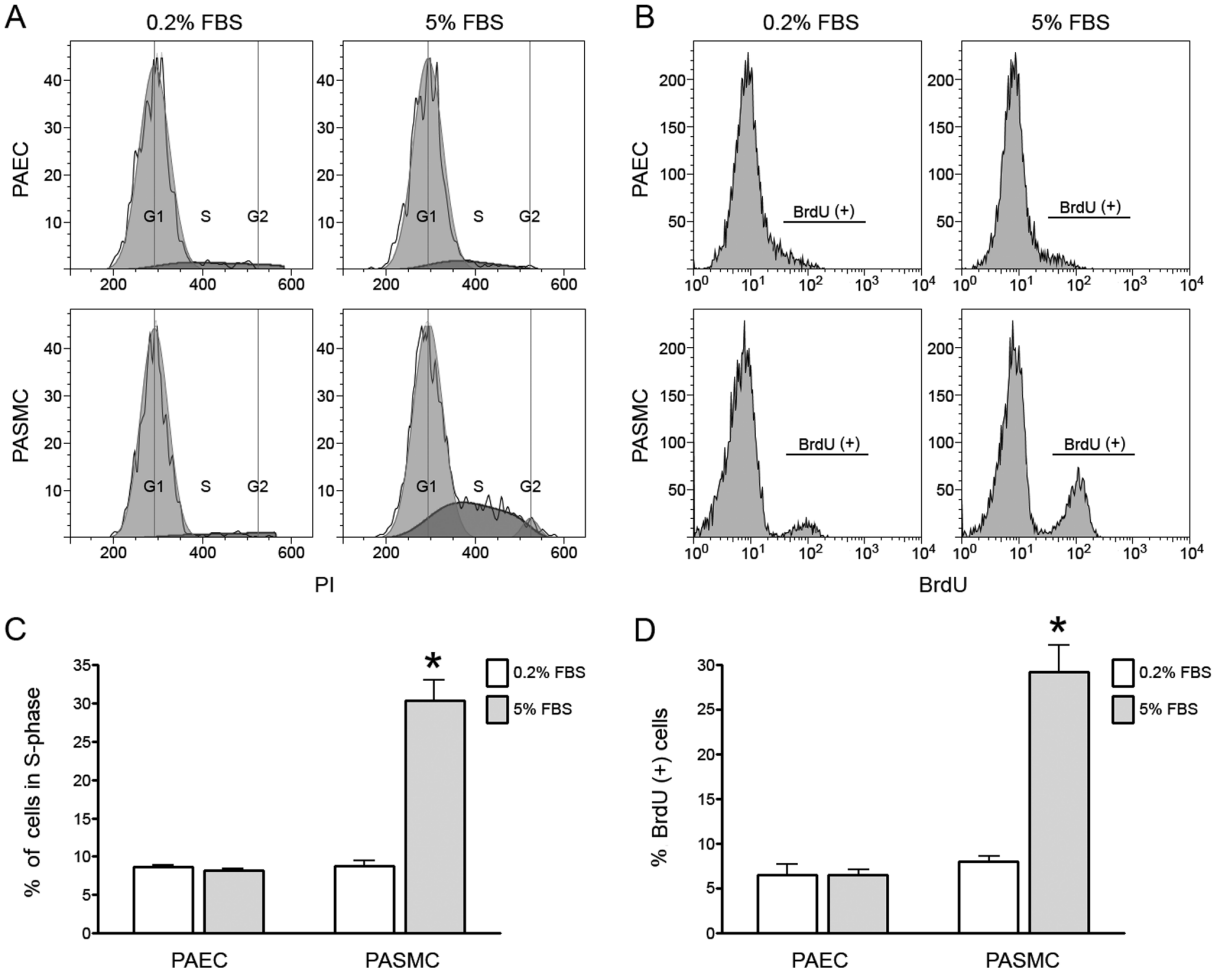
Increased serum stimulates cell cycle progression/DNA synthesis in confluent PASMC, but not PAEC. Human PAEC and PASMC attained confluence in low (0.2%) serum and were maintained in low serum for seven days. Cells were then exposed to 0.2% or 5% serum and cell cycle profile (A and C) and BrdU incorporation (B and D) determined 24 hours later. (n = 3 experiments; * indicates p<.05).

Human PAEC or PASMC were grown in medium with 5% FBS until 90% confluent and then in 0.2% FBS for 6 more days until 100% confluent. Confluence was determined both by light microscopy and also by demonstrating that cell number for both cell types remained stable in 0.2% serum over time. Cells were then changed into full growth media with different concentrations of FBS ranging from 0.1% to 5%. Cell growth was determined over 7 days. Figure 1 demonstrates that confluent PAEC did not increase in number over seven days even in 5% FBS. In contrast, confluent PASMC proliferated when exposed to higher FBS in a dose-dependent fashion. In a separate experiment, BrdU incorporation and changes in cell cycle profile were determined 24 hours after media was replaced with 5% FBS or fresh 0.2% FBS. Consistent with the results in the previous experiment, confluent PASMC exposed to 5% FBS had a significant increase in the percentage of cells in S phase (Figures 2A and 2C) and in BrdU incorporation (Figures 2B and 2D) when compared to similar cells in 0.2% FBS. In contrast, confluent PAEC demonstrated no such increase when switched from 0.2% to 5% FBS (Figures 2A–2D).

### Serum Inactivates Retinoblastoma and Activates AKT in Confluent PASMC, but not PAEC

**Figure 3:**
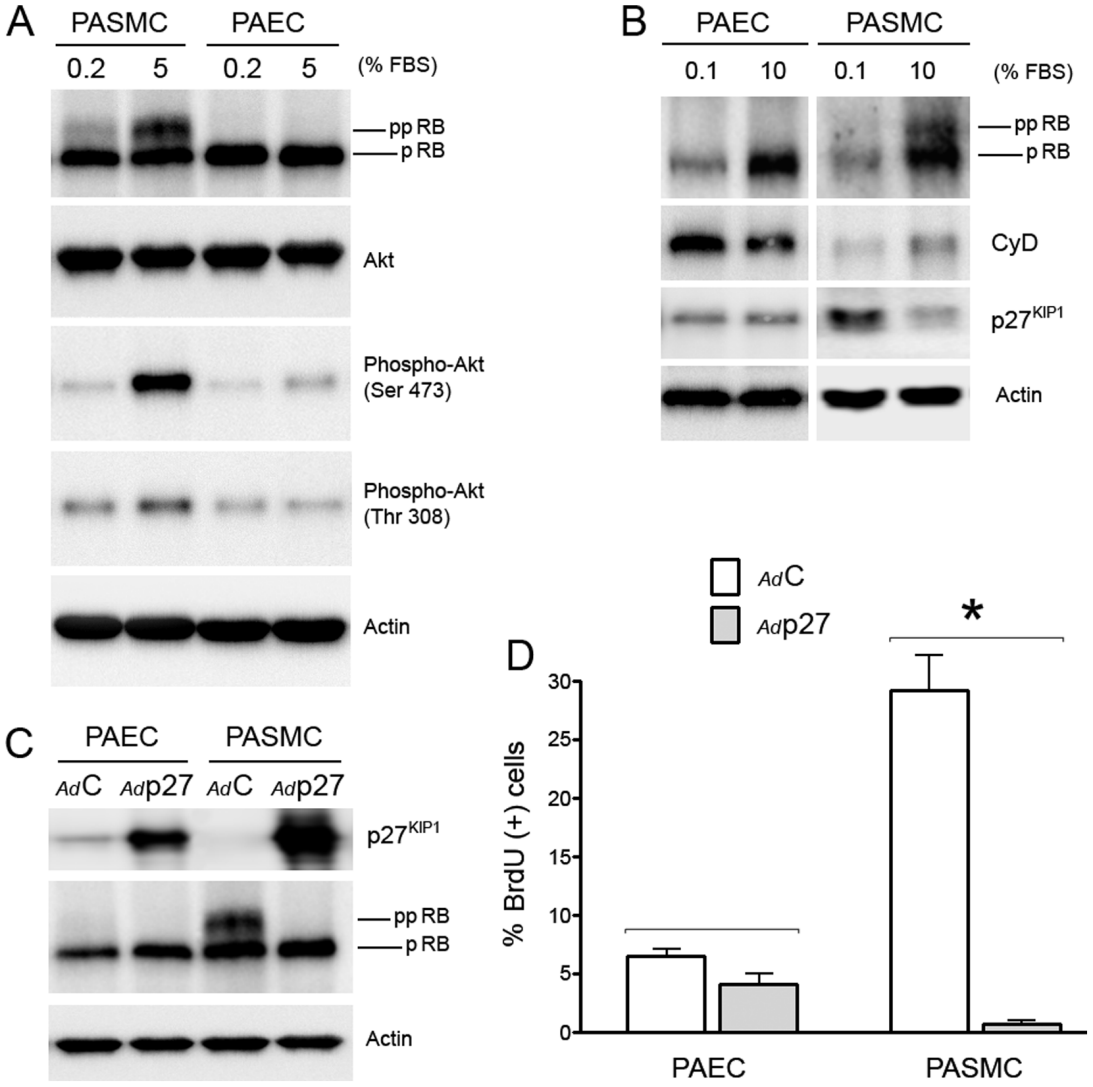
Increased serum phosphorylates retinoblastoma, Akt^Ser473^, and AKT^Thr308^ and decreases p27^KIP1^ in confluent PASMC, but not in confluent PAEC. A and B) Human PAEC and PASMC attained confluence in low (0.2%) serum and were maintained in low serum for seven days. Cells were then exposed to 0.2% or 5% serum and 24 hours later, cell lysates were harvested for protein analysis. Representative Western blots are shown. C) Human PAEC and PASMC attained confluence in low (0.2%) serum and were maintained in low serum for seven days. Cells were infected for 2 hours at a multiple of infectivity of 200 with a replication-deficient adenovirus serotype 5 containing either a human p27^KIP1^ (*Ad*p27) or alkaline phosphatase (*Ad*C) cDNA driven by a CMV promoter. Cells were then exposed to 5% serum and cell lysates harvested 24 hours later. A representative Western blot is shown. [pRB: hypophosphorylated retinoblastoma; ppRB: hyperphosphorylated retinoblastoma]; D) BrdU incorporation 24 hours after exposure to 5% serum in p27^KIP1^-infected human PAEC and PASMC.

Confluent cells were growth arrested in 0.2% FBS for 7 days, exposed to 5% FBS for 24 hours and cell lysates harvested for protein analysis. Figure 3A demonstrates the increase in hyperphosphorylated (inactive) retinoblastoma indicating that stimulated human PASMC had traversed the restriction point of G_1_ and entered S phase whereas human PAEC remained in G_0_/G_1_. Since activation of AKT (protein kinase B) has been demonstrated to be important in mediating proliferative and survival signals in cells [14], we examined AKT phosphorylation in the 2 cell types under different serum conditions. Figure 3A demonstrates that confluent PAEC did not, whereas confluent PASMC did, increase phosphorylation of AKT on threonine 308 and serine 473 in response to serum. We then examined the effect of an increase in serum concentration on expression of the G_1_ cyclin dependent kinase inhibitor, p27^KIP1^. Figure 3B demonstrates that following exposure to 5% serum, p27^KIP1^ protein expression is decreased and cyclin D1 expression is increased in confluent human PASMC, but not in PAEC. Overexpression of p27^KIP1^ in PASMC (using a replication-deficient adenovirus) prior to increasing the serum concentration prevented retinoblastoma hyperphosphorylation (Figure 3C) and inhibited BrdU incorporation (Figure 3D).

**Figure 4:**
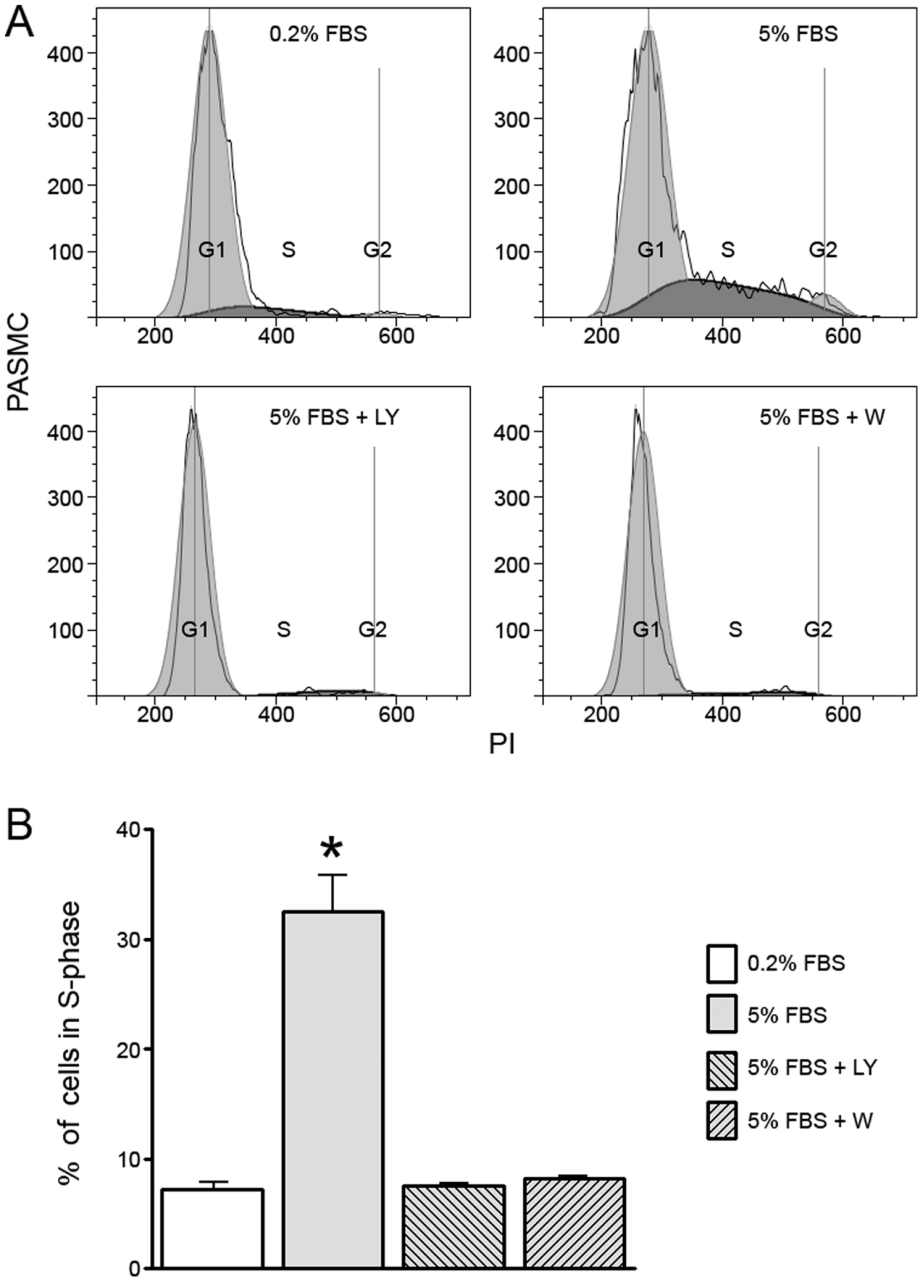
The PI3-kinase inhibitors, LY294002 and wortmannin, block serum-induced proliferation in confluent PASMC. Human PASMC attained confluence in low (0.2%) serum and were maintained in low serum for seven days. Cells were then exposed to 0.2% or 5% serum in the presence or absence of the PI3-kinase inhibitors LY294002 (15 µmol) or wortmannin (50 nmol) and cell cycle profile determined 24 hours later. A) Representative plot of single experiment; B) Aggregate results from 3 separate experiments (n = 3 experiments, * indicates p<.05).

**Figure 5:**
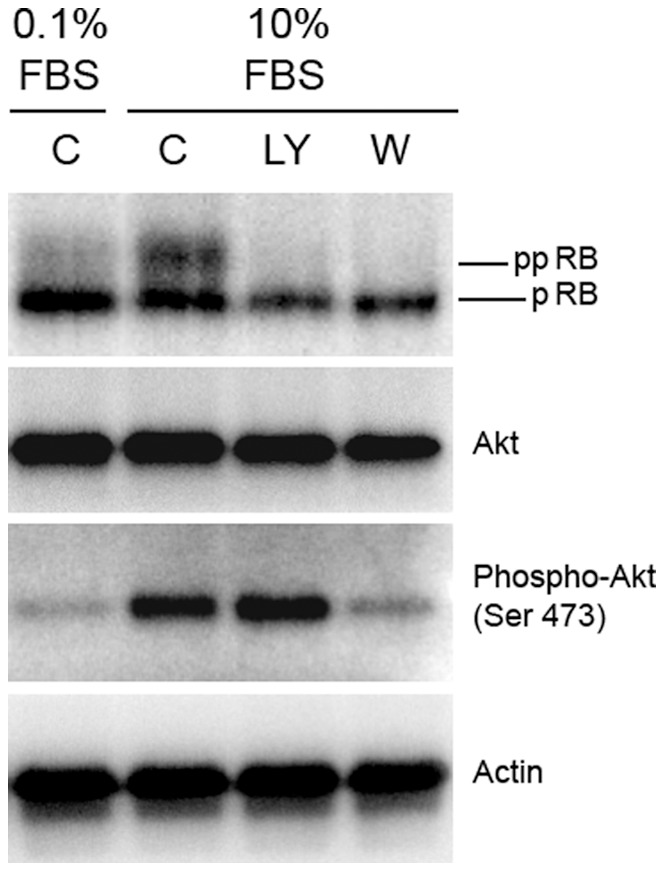
PI3-kinase inhibition prevents retinoblastoma hyperphosphorylation in confluent PASMC exposed to high serum concentrations. Human PASMC attained confluence in low (0.2%) serum and were maintained in low serum for seven days. Cells were then exposed to 0.1% or 5% serum in the presence or absence of LY294002 or wortmannin and cell lysates harvested 24 hours later for protein analysis. Representative Western blot is shown. [pRB: hypophosphorylated retinoblastoma; ppRB: hyperphosphorylated retinoblastoma].

**Figure 6:**
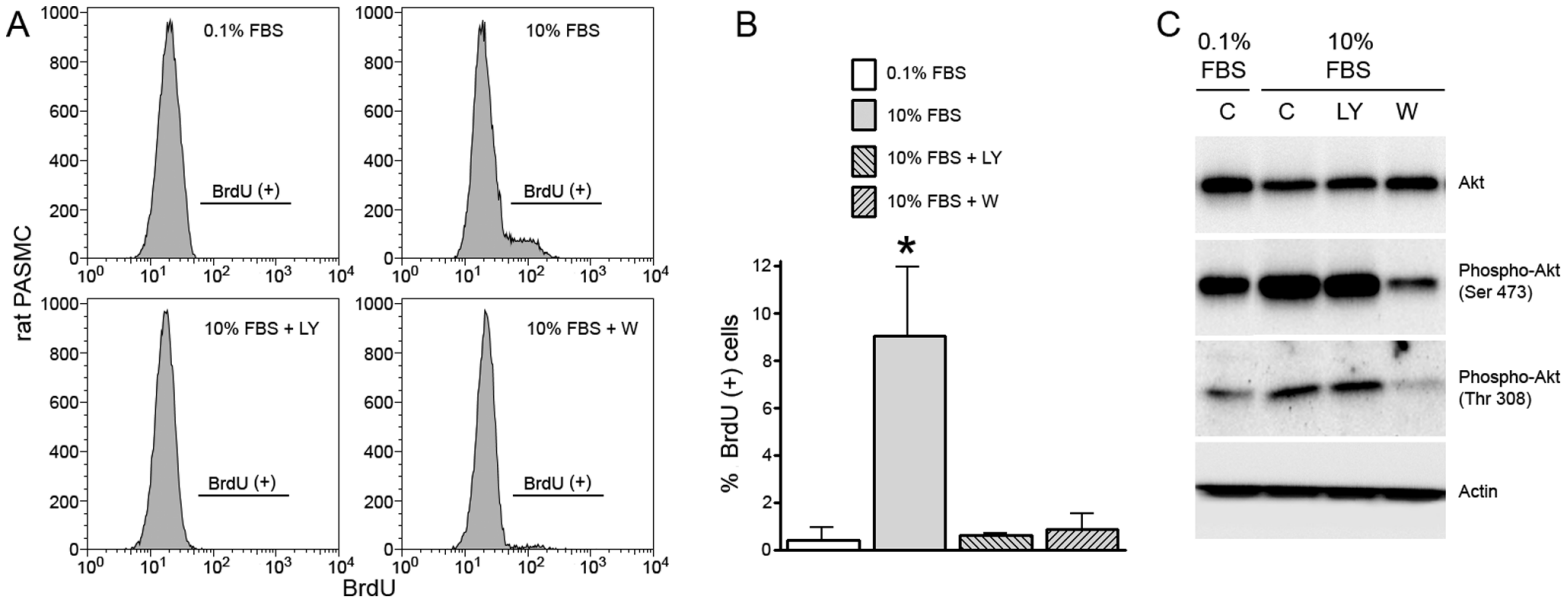
PI3-kinase inhibitors, LY294002 and wortmannin, inhibit rat PASMC proliferation, but only wortmannin inhibits AKT phosphorylation. Rat PASMC were grown to confluence, growth arrested in 0.1% serum for 72 hours, and then exposed to 10% or fresh 0.1% FBS in the presence or absence of wortmannin (W) or LY294002 (LY). Cells were examined 24 hours later for BrdU incorporation (A and B) and AKT phosphorylation (C), (n = 3 experiments, * indicates p<.05).

Since it appeared that serum activated AKT in confluent PASMC, we examined the effect of blocking phosphoinositide 3-kinase (PI3-kinase) activation on cell cycle progression and BrdU incorporation using the inhibitors, LY294002 and wortmannin. Figure 4 demonstrates that both wortmannin and LY294002 blocked serum-induced PASMC proliferation as evidenced by a reduction of cells in S phase. Consistent with this effect, both agents blocked the hyperphosphorylation of retinoblastoma following exposure to 5% FBS (Figure 5). Closer examination of LY294002’s and wortmannin’s effect on AKT phosphorylation suggested that these inhibitors were working in different ways to block PASMC proliferation, however. Wortmannin blocked phosphorylation of AKT^ser473^ whereas LY294002 did not. We confirmed this observation in rat PASMC (Figure 6) indicating that this was not just a species-specific phenomenon. Therefore, while both of these PI3-kinase inhibitors blocked serum-induced proliferation of PASMC, they appeared to do it through different mechanisms that converged at the G_0_/G_1_-S transition resulting in the maintenance of retinoblastoma in its hypophosphorylated, growth-suppressive, state.

### PASMC Density is Determined by Serum Concentration

**Figure 7:**
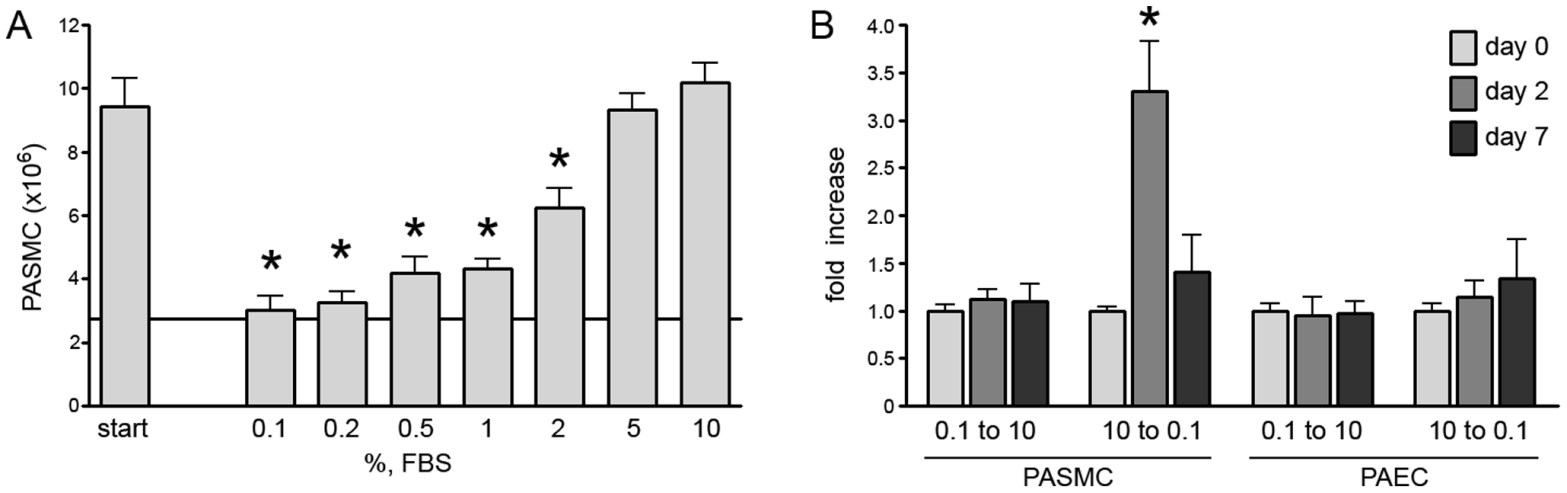
PASMC that attain confluence at high serum cannot maintain that cell density in low serum. Human PASMC were grown to confluence in 10% FBS and maintained in 10% serum for 7 days. Confluence was determined by light microscopy and by the demonstration of stable cell number over 7 days. Cells were then exposed to various concentrations of serum ranging from 0.1% to 10%: A) Cell counts done 7 days later. B) Cells were stained with the membrane impermeable dye ethidium homodimer-1 at different time points to determine cell viability. Ethidium homodimer-1 can only stain apoptotic or necrotic cells and is a marker of cell death. Cell death is increased by day 2 and returns to baseline by day 7. (n = 3 separate experiments, * indicates p<.05 compared with starting cell number for (A) or compared to day 0 at 10% serum for (B).

Our results in Figure 1 indicated that when confluent PASMC were exposed to increasing concentrations of serum, they proliferated to a higher density, a density determined by the concentration of serum. We next examined whether this increased density would be sustained if the serum levels were decreased. PASMC were grown in 5% serum until confluent. Cells were then maintained in 5% serum for an additional 7 days to confirm that proliferation had ceased, and then switched back to lower serum concentrations; cell number was determined seven days later. A reduction in serum concentration decreased cell number and after seven days in 0.2% serum, PASMC returned to the (lower) density at which confluence was initially attained (Figure 7A). Staining the PASMC culture with Ethidium homodimer-1, a dye impermeable to living cells, during serum reduction demonstrated scattered cell death throughout the plate that peaked at day 2 and subsided once the lower density was re-established after seven days (Figure 7B).

## Discussion

This paper demonstrates that once PAEC attain confluence, further proliferation is inhibited, whereas contact inhibition in PASMC can be overcome by exposure to a higher concentration of serum. We also demonstrate that once the higher concentration of serum is removed, PASMC return to a lower density, a density that correlates with that attained during initial confluence. We identify the ability of acute increases in serum to inactivate (hyperphosphorylate) the tumor suppressor, retinoblastoma, in confluent pulmonary artery smooth muscle cells, but not in confluent pulmonary artery endothelial cells, in a PI3-kinase-dependent pathway, as a potential explanation for this observation.

Multiple pathways important in mediating contact inhibition have been identified in different cell types and under different conditions. [1]–[6], [15], [16] Whether these different pathways are exclusive to a particular cell type or whether multiple pathways may be active in a cell at any time is not clear. In addition, it is not clear whether any particular pathway may be more effective than another at inducing contact inhibition in the face of continuous exposure to growth factors. In both the systemic and pulmonary circulation, endothelial cells are in continuous contact with circulating growth factors and therefore require strict contact inhibition to avoid pathologic vascular proliferation. Inhibition of p42/p44 MAPK activity, [7] upregulation of protein tyrosine kinase phosphatases leading to dephosphorylation of receptor tyrosine kinases and adherens junction proteins, [16] transcriptional upregulation of the cyclin dependent kinase inhibitor p27^Kip1^, [5] and the mobilization of cholesterol to the cell membrane as cells near confluence, [2] are some of the factors identified as important mediators of contact inhibition in endothelial cells.

Vascular smooth muscle cells, in contrast, are not normally exposed to serum. They come to confluence in low (or no) serum and are maintained in this same low serum state in health. Mechanisms to maintain contact inhibition upon exposure to high levels of growth factors may be less effective than those in endothelial cells. In a paper examining the effect of growth arrest on the efficiency of gene transfer, Pelisek and colleagues demonstrated that porcine aortic smooth muscle cells did not completely come out of cycle at confluence in 10% serum whereas endothelial cells did. [17] The addition of heparin to media was effective in inducing growth arrest in confluent SMC indicating that SMC could be growth arrested, but that confluence was not a sufficiently strong stimulus to do this across an entire monolayer. Our results confirm, in human cells from the pulmonary circulation, the relative ineffectiveness of confluence to inhibit proliferation in vascular smooth muscle cells compared to endothelial cells.

In addition, our results indicate that the ability of confluent PASMC, but not confluent PAEC, to proliferate upon exposure to higher concentrations of serum was due, at least in part, to differences in PI3-kinase activation. Two PI3-kinase inhibitors, wortmannin and LY294002, prevented PASMC proliferation during exposure to 5% FBS as indicated by a decrease in BrdU incorporation and a reduction in the percentage of cells in S phase. Despite similar inhibitory effects on cell proliferation, however, the two inhibitors had different effects on AKT phosphorylation. While both were able to prevent the hyperphosphorylation (inactivation) of the tumor suppressor, retinoblastoma, LY294002 did not block the phosphorylation of AKT^ser473^ or AKT^Thr308^, whereas wortmannin did. We confirmed these disparate effects of LY294002 and wortmannin in rat PASMC indicating that this was not just a species-specific phenomenon.

LY294002’s inability to block AKT phosphorylation was surprising, but reproducible, even in doses three-fold higher than its IC_50_ for PI3-kinase. Both inhibitors localize to the ATP binding pocket of PI3-kinase, but do so with different affinities and different effects on conformation of the enzyme. [18], [19] LY294002 is a competitive inhibitor [20] whereas wortmannin binds irreversibly to the site with an IC_50_ in the nanomolar range, approximately 3 logs lower than LY294002’s IC_50_. [18] In addition, there are multiple isoforms of PI3-kinase and the two inhibitors have different affinities for each.[21]–[23] LY294002 and wortmannin, therefore, may have different effects on downstream PI3-kinase signaling pathways, each of which may be necessary to hyperphosphorylate retinoblastoma and allow cells to enter S phase. Whatever the reason, the ability of both LY294002 and wortmannin to block PASMC proliferation despite the observation that only wortmannin inhibited AKT phosphorylation, suggests that serum’s ability to stimulate confluent PASMC involves PI3-kinase signaling pathways other than AKT. In addition, other intracellular pathways such as MAP kinase [7], [24], [25] and Rho/Rho kinase [26]–[28] are known inducers of cell proliferation and activation of them in confluent human PASMC, but not PAEC in response to increased serum, may have contributed to these observations as well. Blocking the hyperphosphorylation of retinoblastoma with either LY294002, wortmannin, or with overexpression of the cyclin dependent kinase inhibitor, p27^KIP1^, prevented cell proliferation suggesting a process that converges at the level of the cell cycle.

Once confluent, both PAEC and PASMC stopped proliferating and could be maintained in minimal serum. Only PASMC proliferated beyond confluence, and only if exposed to a higher concentration of serum than that present at initial confluence. The final density of PASMC was determined by the concentration of serum in the media, higher concentrations of serum yielded greater numbers of PASMC. This higher density could only be maintained, however, if cells remained exposed to high levels of serum. Once the serum concentration was decreased, PASMC numbers returned to a level that correlated with their initial growth density at that same serum concentration. If serum levels were returned all the way back to 0.2%, PASMC density decreased to that attained at initial confluence. To accomplish this, PASMC underwent cell death that peaked around day two and then decreased to baseline by day seven. These results suggest that events that expose PASMC to serum such as injury to the vascular endothelium, or transient increases in its permeability, can lead to smooth muscle cell proliferation, but that once the vascular leak resolves, and smooth muscle cells are no longer exposed to high levels of serum, this hyperplasia can be reversed.

Pulmonary artery smooth muscle cell proliferation is an important pathophysiologic event in the development of pulmonary arterial hypertension. [10] Endothelial injury is common in many animal models of pulmonary hypertension [8]–[10] and also in humans with idiopathic pulmonary arterial hypertension. [29] Both laminar and turbulent flow, common in pulmonary hypertension, caused endothelial injury sufficient to allow extravasation of Evan’s Blue dye after just one hour in an animal model of partial vascular occlusion. [30] Monocrotaline, a toxicant used to induce pulmonary hypertension in animals, causes a significant endothelial injury leading to hyperplasia and hypertrophy of the underlying smooth muscle. [8] These effects of monocrotaline on smooth muscle were not likely due to the direct toxic effects of monocrotaline (or more accurately, of its toxic metabolite, monocrotaline pyrrole), however, since smooth muscle cell monolayers in culture were neither injured nor stimulated to proliferate by direct exposure to monocrotaline pyrrole in contrast to monocrotaline’s marked detrimental effects on cultured endothelial cells. [31] In animal models of hypoxia-induced pulmonary hypertension, PASMC proliferation is an important histological finding that peaks within the first week of hypoxic exposure based on BrdU incorporation. [10] While the mechanism of hypoxia-induced PASMC proliferation is thought to be due mainly to mechanical forces related to hypoxic pulmonary vasoconstriction, acute hypoxia can lead to an increase in endothelial permeability [32]–[34] suggesting that exposure of the underlying PASMC to serum, may be an important stimulus for the medial hyperplasia and neomuscularization seen in hypoxia-induced pulmonary hypertension.

In summary, we have demonstrated that human pulmonary artery endothelial cells have a significantly stronger level of contact inhibition than the smooth muscle cells that underlie them. The only stimulus that could consistently induce endothelial cells to proliferate in our experiments was loss of confluence. In contrast, vascular smooth muscle cells were able to proliferate when exposed to growth factors or serum even when confluent. This suggests that stimuli that increase endothelial permeability can lead to vascular smooth muscle cell hyperplasia and that this hyperplasia can persist as long as exposure to serum remains. Our data suggest that once vascular smooth muscle cells are returned to low serum levels, as might be expected to occur following repair of endothelial injury, medial hyperplasia should be reversed and the initial (pre-injury) density of cells restored through cell death. The observation that smooth muscle cell hyperplasia does not always reverse clinically, even when endothelial barrier integrity appears to be restored, suggests that other proliferative stimuli, such as autocrine/paracrine release of growth factors or other, non-growth factor stimuli such as increased shear stress, persist.
